# Uncertainty propagation in pore water chemical composition calculation using surrogate models

**DOI:** 10.1038/s41598-022-18411-5

**Published:** 2022-09-05

**Authors:** Pierre Sochala, Christophe Chiaberge, Francis Claret, Christophe Tournassat

**Affiliations:** 1 CEA, DAM, DIF, 91297 Arpajon, France; 2 BRGM, 3 avenue Claude Guillemin, 45060 Orléans, France; 3 ISTO, Université d’Orléans-CNRS-BRGM, Orléans, France; 4 Lawrence Berkeley National Laboratory, Berkeley, CA USA

**Keywords:** Computational science, Biogeochemistry

## Abstract

Performance assessment in deep geological nuclear waste repository systems necessitates an extended knowledge of the pore water chemical conditions prevailing in host-rock formations. In the last two decades, important progress has been made in the experimental characterization and thermodynamic modeling of pore water speciation, but the influence of experimental artifacts and uncertainties of thermodynamic input parameters are seldom evaluated. In this respect, we conducted an uncertainty propagation study in a reference geochemical model describing the pore water chemistry of the Callovian-Oxfordian clay formation. Nineteen model input parameters were perturbed, including those associated to experimental characterization (leached anions, exchanged cations, cation exchange selectivity coefficients) and those associated to generic thermodynamic databases (solubilities). A set of 13 quantities of interest were studied by the use of polynomial chaos expansions built non-intrusively with a least-squares forward stepwise regression approach. Training and validation sets of simulations were carried out using the geochemical speciation code PHREEQC. The statistical results explored the marginal distribution of each quantity of interest, their bivariate correlations as well as their global sensitivity indices. The influence of the assumed distributions for input parameters uncertainties was evaluated by considering two parametric domain sizes.

## Introduction

Knowledge of pore water chemical composition is crucial for the building of nuclear waste repository performance assessments^1^. First, pore water chemical composition controls radionuclides solubility and adsorption properties on geological and engineered materials. Second, pore water chemical composition influences the transport and mechanical properties of clayey materials, which are essential constituents of existing multi-barrier concepts^2^. Third, pore water chemical composition dictates the nature and kinetics of chemical alteration processes of repository exogenous materials, such as concrete and nuclear glass^3^. But pore water chemical composition models contain a significant number of input parameters, exhibit strong nonlinearities, and have tightly coupled output results. For these reasons, it is difficult to estimate uncertainties on each of the input parameters and to evaluate the uncertainties of the model outputs, using e.g. error propagation methods. A direct sampling of clay pore water that retains the main characteristics representative of in situ conditions is particularly complex because of a range of side reactions taking place during sampling procedures^4^. Consequently, confidence in the knowledge of pore water chemical composition must be built on a consistent combination of several factors, which includes in situ seepage water collection and characterization, experimental water rock interactions results, and geochemical modeling results linking observations about solid material composition and reactivity with quantitative thermo-kinetic concepts^5^. While considerable effort with this experimental and modeling coupled approach enabled to produce predictive models for claystone pore water chemical composition that are consistent with experimental characterization, little attention has been directed to evaluate the model output uncertainties induced by input parameters uncertainties. Indeed, although the treatment of uncertainties in the performance assessment of geologic high-level radioactive waste repositories is recognized as an important topic for more than three decades^6^, most of the studies focus on uncertainties related to retention processes^7^. Using Monte Carlo methods, the effects of database parameter uncertainty have been evidenced on geochemical equilibrium calculations but limited to very simple (as stated by the authors) modeling scenarios^8^. The goal of the present study is the implementation of a methodology based on surrogate models designed to propagate parametric uncertainties into a pore water chemical composition model with a moderate number of input parameters (around twenty).

Propagation of uncertainty gained wide popularity in many geosciences disciplines^9–12^. Its principle consists in perturbing a set of input parameters and then estimating the ensuing effects on the output quantities. The interest of such statistical framework is to produce richer and more useful information than a single deterministic simulation can deliver. Parametric uncertainty analyses in geochemistry are motivated by different sources of uncertainty such as reaction kinetic rate constants, thermodynamic constants (e.g. solubility and aqueous complex formation constants), initial and boundary conditions, and transport properties^13–16^. Among available approaches, surrogate models have the advantage of providing a fast approximation everywhere in the parametric domain from a small ensemble of simulations, whereas Monte-Carlo techniques evaluate the direct model for a finite number of samples and require a large ensemble to achieve the convergence of the statistical estimators.

In this study, we are interested in using a surrogate model approach to propagate uncertainty into a pore water chemical composition model of the Callovian-Oxfordian (COx) clay formation in the Paris Basin (France), which has been the target of many studies investigating the feasibility of deep nuclear waste repository^17^. First, we briefly summarize the geochemical model and the parametric domain on which statistical approximations of the different quantities of interest (QoI) were built. Second, we describe the construction and validation of the surrogate models, with a Polynomial Chaos (PC) method and an orthogonal matching pursuit procedure, which are particularly efficient if the QoI exhibit smooth variations when the uncertain inputs vary. At last, we focus discussion on moments, marginal distributions, correlations and joint distributions as well as on global sensitivity indices, which quantify the influence of the input parameter distributions onto the variance of the QoIs.

## Framework

### Pore water composition model

The estimation of pore water chemical composition in the COx claystone relies on a geochemical model, of which complete description can be found in^4^. The model is briefly presented and made available in the form of a PHREEQC^18^ input file and its associated database (THERMOCHIMIE v9b^19^) in the supplementary information file. The complete list of pore water chemical composition model input parameters are: ${\mathrm{Cl}}^{-}$ and ${\mathrm{SO}}_{4}^{2-}$ total concentration obtained from core sample leaching measurements; measured sodium ${\mathrm{Na}}^{+}$, potassium ${\mathrm{K}}^{+}$, calcium ${\mathrm{Ca}}^{2+}$, magnesium ${\mathrm{Mg}}^{2+}$, and strontium ${\mathrm{Sr}}^{2+}$ exchangeable concentrations; related ${\mathrm{Na}}^{+}/{\mathrm{K}}^{+}$, ${\mathrm{Na}}^{+}/{\mathrm{Ca}}^{2+}$, ${\mathrm{Na}}^{+}/{\mathrm{Mg}}^{2+}$, ${\mathrm{Na}}^{+}/{\mathrm{Sr}}^{2+}$ cation exchange selectivity coefficients; and solubilities of Celestite, Calcite, Dolomite, Goethite, Quartz, Pyrite, Ripidolite, and Illite (corresponding to illite$\_$Imt-2 of the database). The reference values of these $N=19$ parameters are reported in Table 1.

**Table 1:** List of the 19 uncertain input parameters with their reference values $\mu$ (unperturbed state of the geochemical model).

$\#$	Type	Species	Unit	$\mu$
1	Leached parameter	${\mathrm{Cl}}^{-}$	${\mathrm{mmol}}\; {\mathrm{L}}^{-1}$	41
2		${\mathrm{SO}}_{4}^{2-}$	${\mathrm{mmol}}\; {\mathrm{L}}^{-1}$	66
3	Exchanged cation	${\mathrm{Na}}^{+}$	${\mathrm{mol}}\; {\mathrm{L}}^{-1}$	1.0824
4		${\mathrm{K}}^{+}$	${\mathrm{mol}}\; {\mathrm{L}}^{-1}$	0.417
5		${\mathrm{Ca}}^{2+}$	${\mathrm{mol}}\; {\mathrm{L}}^{-1}$	1.549
6		${\mathrm{Mg}}^{2+}$	${\mathrm{mol}}\; {\mathrm{L}}^{-1}$	0.602
7		${\mathrm{Sr}}^{2+}$	${\mathrm{mol}}\; {\mathrm{L}}^{-1}$	0.0737
8	Selectivity coefficients ($\log K_{\mathrm{ex}}$ value)	${\mathrm{Na}}^{+}/{\mathrm{K}}^{+}$	–	1.2
9		${\mathrm{Na}}^{+}/{\mathrm{Ca}}^{2+}$	–	0.7
10		${\mathrm{Na}}^{+}/{\mathrm{Mg}}^{2+}$	–	0.7
11		${\mathrm{Na}}^{+}/{\mathrm{Sr}}^{2+}$	–	0.6
12	Solubility ($\log K$ value)	Celestite	–	$-6.62$
13		Calcite	–	$-8.48$
14		Dolomite	–	$-17.12$
15		Goethite	–	0.39
16		Quartz	–	$-3.74$
17		Pyrithe	–	$-58.78$
18		Ripidolite	–	61.35
19		Illite	–	11.54

### Uncertainty model

Once the uncertain input parameters have been identified, the next step is to determine their statistical distributions. For a scalar parameter, it consists of specifying a range (or support) and an associated probability density function. The *N* uncertain inputs were collected into a random vector ${\varvec{\xi }}=(\xi _1,\dots ,\xi _N)\in {\varvec{\Xi }}\subset \mathbb {R}^N$ whose components $\xi _i$ were assumed to be independent and uniformly distributed over the range $[\xi _i^{-},\xi _i^{+}]$, namely

1 $$\begin{aligned} \xi _i \sim \mathscr {U}\left( \xi _i^{-},\xi _i^{+}\right) ,\quad \xi _i\perp \xi _j\quad \text {if}\quad i\ne j. \end{aligned}$$

The assumption of independence implies that the joint distribution $p_{{\varvec{\xi }}}$ of the vector ${\varvec{\xi }}$ and therefore its range ${\varvec{\Xi }}$ factorizes to

2 $$p_{\boldsymbol{\xi}} (\boldsymbol{\xi}) = \prod_{i=1}^N p_{\xi_i}(\xi_i;\xi_i^{-},\xi_{i}^{+})\quad\text{and}\quad {\boldsymbol{\Xi}}=\prod_{i=1}^N \left[\xi_i^{-},\xi_i^{+}\right].$$

In case of a uniform distribution, the probability density function of each parameter $\xi _i$ is defined as

3 $$\begin{aligned} p_{\xi _i}(\xi _ i;\xi _i^{-},\xi _i^{+}):= {\left\{ \begin{array}{ll} 1/(\xi _i^{+}-\xi _i^{-}), &{} \xi _i\in \left[ \xi _i^{-},\xi _i^{+}\right] , \\ 0, &{} \text {otherwise}.\\ \end{array}\right. } \end{aligned}$$

The extreme values $\xi _i^{-}$ and $\xi _i^{+}$ of the *i*th parameter are defined as

4 $$\begin{aligned} \xi _i^{-}=\mu _i-\sqrt{3}\sigma _i\quad \text {and}\quad \xi _i^{+}=\mu _i+\sqrt{3}\sigma _i, \end{aligned}$$

where the mean $\mu _i=\mathbb {E}(\xi _i)$ corresponds here to the reference value indicated in Table 1 and the standard deviation $\sigma _i=\sqrt{\mathbb {V}(\xi _i)}$ is reported in Table 2. Recall that the mean $\mathbb {E}(\cdot )$ and the variance $\mathbb {V}(\cdot )$ of a random variable *u* are defined as

5 $$\begin{aligned} \mathbb {E}(u)&:=\int _{\Xi }u({\varvec{\xi }})p_{{\varvec{\xi }}}({\varvec{\xi }})d{\varvec{\xi }}, \end{aligned}$$

6 $$\begin{aligned} \mathbb {V}(u)&:=\mathbb {E}\left[ (u-\mathbb {E}(u))^2\right] . \end{aligned}$$

We have chosen the uniform distribution since it is the maximum entropy distribution^20,21^ among all continuous distributions which are supported in a given finite range. The maximum entropy distribution is often preferred because it represents the least informative distribution but other types of distributions can be adopted. Two cases were considered in order to investigate the effect of the amplitude of perturbations around the reference values onto the uncertainty of the QoIs. Hereafter, these cases are referred to as the “small range case” and “large range case”, respectively. Each parameter range of the latter case is twice the range of the former case, implying from Eq. (2) that the measure (or area) of the parametric domain ${\varvec{\Xi }}$ in the large range case is $2^{N}\simeq 5\cdot 10^5$ times higher than in the small range case.

**Table 2:** Standard deviation $\sigma$, minimal value $\xi ^{-}$ and maximal value $\xi ^{+}$ of the 19 uncertain input parameters for the small range case and the large range case.

Species	Small range case			Large range case		
	$\sigma$	$\xi ^{-}$	$\xi ^{+}$	$\sigma$	$\xi ^{-}$	$\xi ^{+}$
${\mathrm{Cl}}^{-}$	$2.05\ (5\%)$	37.4	44.6	$4.10\ (10\%)$	33.9	48.1
${\mathrm{SO}}_{4}^{2-}$	$3.30\ (5\%)$	60.3	71.7	$6.60\ (10\%)$	54.6	77.4
${\mathrm{Na}}^{+}$	$0.05\ (5\%)$	0.99	1.17	$0.1\ (10\%)$	0.91	1.25
${\mathrm{K}}^{+}$	$0.02\ (5\%)$	0.38	0.45	$0.04\ (10\%)$	0.35	0.49
${\mathrm{Ca}}^{2+}$	$0.08\ (5\%)$	1.41	1.69	$0.16\ (10\%)$	1.27	1.83
${\mathrm{Mg}}^{2+}$	$0.03\ (5\%)$	0.55	0.65	$0.06\ (10\%)$	0.50	0.70
${\mathrm{Sr}}^{2+}$	$0.04\ (5\%)$	0.067	0.081	$0.08\ (10\%)$	0.06	0.088
${\mathrm{Na}}^{+}/{\mathrm{K}}^{+}$	$0.1\ (8\%)$	1.03	1.37	$0.2\ (16\%)$	0.85	1.55
${\mathrm{Na}}^{+}/{\mathrm{Ca}}^{2+}$	$0.1\ (14\%)$	0.53	0.87	$0.2\ (28\%)$	0.35	1.05
${\mathrm{Na}}^{+}/{\mathrm{Mg}}^{2+}$	$0.1\ (14\%)$	0.53	0.87	$0.2\ (28\%)$	0.35	1.05
${\mathrm{Na}}^{+}/{\mathrm{Sr}}^{2+}$	$0.1\ (17\%)$	0.43	0.77	$0.2\ (34\%)$	0.25	0.95
Celestite	0.05	$-6.71$	$-6.53$	0.1	$-6.79$	$-6.45$
Calcite	0.05	$-8.57$	$-8.39$	0.1	$-8.65$	$-8.31$
Dolomite	0.2	$-17.5$	$-16.8$	0.4	$-17.8$	$-16.4$
Goethite	0.2	0.044	0.74	0.4	$-0.30$	1.08
Quartz	0.05	$-3.83$	$-3.65$	0.1	$-3.91$	$-3.57$
Pyrite	0.2	$-59.1$	$-58.4$	0.4	$-59.5$	$-58.1$
Ripidolite	0.5	60.5	62.2	1	59.6	63.1
Illite	0.5	10.7	12.4	1	9.81	13.3

### Quantities of interest

We are interested in $\mathrm{pH}$, $\mathrm{pe}+\mathrm{pH}$ where $\mathrm{pe}$ is the redox potential, total aqueous concentrations of sodium (${\mathrm{Na}}^{+}$), potassium (${\mathrm{K}}^{+}$), calcium (${\mathrm{Ca}}^{2+}$), magnesium (${\mathrm{Mg}}^{2+}$), strontium (${\mathrm{Sr}}^{2+}$), iron ($\mathrm{Fe}$), silicium ($\mathrm{Si}$), aluminum ($\mathrm{Al}$), sulphate ($\mathrm{S(VI)}$) and sulfur ($\mathrm{S(-II)}$), as well as the $\log _{10}$ of ${\mathrm{CO}}_{2}$ partial pressure ${\mathrm{log}}_{10}\,{\mathrm{p}}_{{\mathrm{CO}}_2}$. The set of these $\mathscr {N}=13$ QoI is denoted $\mathbb {U}$,

7 $$\begin{aligned} \mathbb {U}:=\left\{ \mathrm{pH}, \mathrm{pe}+\mathrm{pH}, {\mathrm{Na}}^{+}, {\mathrm{K}}^{+}, {\mathrm{Ca}}^{2+}, {\mathrm{Mg}}^{2+}, {\mathrm{Sr}}^{2+}, \mathrm{Fe}, \mathrm{Si}, \mathrm{Al}, \mathrm{S(VI)}, \mathrm{S(-II)},{\mathrm{log}}_{10}\,{\mathrm{p}}_{{\mathrm{CO}}_2}\right\} . \end{aligned}$$

## Surrogate model

The non-intrusive construction of a surrogate model relies on a training set $\mathscr {X}:=\{{\varvec{\xi }}^{(m)}\}$ that samples the parametric domain. The corresponding outputs were computed using PHREEQC, and we obtained $\mathscr {U}:=\{u^{(m)}:=u({\varvec{\xi }}^{(m)})\}$ for each $u\in \mathbb {U}$. The input-output relations ${\varvec{\xi }}^{(m)}\rightarrow u^{(m)}$ were then exploited to build an approximation of *u* over the whole parametric domain. Several families of methods have been developed over the past decades to construct surrogate models including Gaussian processes^22^ and (possibly deep) neural networks^23^. In this study, in which 19 input parameters were perturbed, we chose polynomial chaos surrogates^24,25^ for their relatively low computational costs of construction in moderate dimensional case. In this section, after a brief description of PC expansions and a short reminder on the least squares method, we present the orthogonal matching pursuit procedure as well as the validation of the surrogate models.

### Polynomial chaos

Any random variable *u* with finite variance can be approximated by a spectral expansion^26,27^ of the form

8 $$\begin{aligned} u^{\mathscr {K}}({\varvec{\xi }}) = \sum _{{\varvec{k}}\in \mathscr {K}}u_{{\varvec{k}}} \phi _{{\varvec{k}}}({\varvec{\xi }}), \end{aligned}$$

where $\{u_{{\varvec{k}}}\}$ is the set of spectral coefficients of $u^{\mathscr {K}}$ and $\{\phi _{{\varvec{k}}}({\varvec{\xi }})\}$ is a complete orthogonal set constituting a basis of $L_2({\varvec{\Xi }},p_{{\varvec{\xi }}})$. The $\phi _{{\varvec{k}}}({\varvec{\xi }})$ are *N*-variate Legendre polynomials for uniform distributions as is the case here. Each multivariate polynomial is defined by an integer-valued multi-index ${\varvec{k}}=(k_1,\ldots ,k_N)\in \mathbb {N}^N$ where $k_i$ is the polynomial degree associated to the *i*th variable $\xi _i$. The truncated PC expansion (8) is then defined using a finite set $\mathscr {K}$ of multi-indices and we denote $N_{\mathrm{b}}:=\left| \mathscr {K}\right|$ the PC basis dimension. Sets of multi-indices are often chosen by prescribing a maximal degree $d^{\circ }$ leading to

9 $$\begin{aligned} {\mathscr {K}}(d^{\circ }) = \left\{ {\varvec{k}}\in \mathbb {N}^N , \left\| {\varvec{k}}\right\| _1 \le d^{\circ }\right\} \quad \text {and}\quad N_{\mathrm{b}}(d^{\circ })=\frac{(N+d^{\circ })!}{N!d^{\circ }!}. \end{aligned}$$

Least squares method is an efficient approach to estimate the spectral coefficients but cannot be applied if the sample size *M* is much lower than the PC basis dimension $N_{\mathrm{b}}$. In this case, more advanced methods are used to produce sparse PC.

### Ordinary least squares

A first way of estimating the spectral coefficients of a PC expansion is to use the Ordinary Least Squares (OLS) method that consists of minimizing the squared norm of the residual,

10 $$\begin{aligned} \min _{{\varvec{u}}}\Vert A {\varvec{u}}-\mathbbm {u}\Vert _2^2, \end{aligned}$$

where $A\in \mathbb {R}^{M,N_{\mathrm{b}}}$ is the matrix of basis functions $\phi _{{\varvec{k}}}({\varvec{\xi }}^{(m)})$, ${\varvec{u}}\in \mathbb {R}^{N_{\mathrm{b}}}$ collects the spectral coefficients $u_{{\varvec{k}}}$ and $\mathbbm {u}\in \mathbbm {R}^{M}$ is the vector of model output $u({\varvec{\xi }}^{(m)})$. The solution of the minimization problem (10) satisfies the system of normal equations

11 $$\begin{aligned} A^\top A {\varvec{u}}= A^\top \mathbbm {u}, \end{aligned}$$

provided that the matrix $A^\top A$ is invertible.

### Orthogonal matching pursuit

When dealing with high dimensional case, sparse approximation theory has been developed for finding solutions to underdetermined linear systems under sparsity constraint. Such parsimonious solutions can be justified by the sparsity-of-effects principle stating that most models are usually dominated by main effects and low-order interactions^28^. This principle is illustrated in PC by sparse expansions in which most of the coefficients are zeroes.

Numerous algorithms have been developed recently for the computation of sparse PC expansions (see^29^ for a review of the existing methods). We relied here on the Orthogonal Matching Pursuit (OMP) method that is a classical greedy algorithm to select a set of active basis functions among a large set (or dictionary) of functions. Initially developed in signal processing^30^, the matching pursuit algorithm starts with an empty approximation and adds sequentially the most correlated basis function to the current residual. The index $\gamma ^{k}$ of the new basis function satisfies (for $k\ge 1$),

12 $$\begin{aligned} \gamma ^{k} = {\mathop {\mathrm{arg\,max}}\limits _j}\left( \left| \mathbbm {d}_j^{\top }\mathbbm {r}^{k-1}\right| \right) , \end{aligned}$$

where $\mathbbm {d}_j\in \mathbbm {R}^M$ is the *j*-th column of the dictionary *D* and $\mathbbm {r}^{k-1}\in \mathbbm {R}^M$ the current residual. The orthogonal version of the method^31^ computes the coefficients of the approximation to ensure that the residual is orthogonal to the span of the active functions,

13 $$\begin{aligned} \left( A^{k}\right) ^\top A^{k} {\varvec{u}}^{k} = \left( A^{k}\right) ^\top \mathbbm {u}, \end{aligned}$$

where $A^{k}\in \mathbb {R}^{M,k}$ is the matrix of the active basis functions at iteration *k*. The OMP method is a least-squares forward stepwise regression approach that can be easily implemented (see Supplementary material for the detailed algorithm). Several criteria are possible to stop the iterations, such as the residual norm or cross-validation errors. Here, we compute every ten iterations (until 1500) the Mean Squared Error (MSE) using a validation set $\mathscr {X}_{*}$ of $M_{*}$ realizations, $\mathrm{MSE} := \sum _{{\varvec{\xi }}\in \mathscr {X}_{*}}\big (u\big ({\varvec{\xi }}\big )-u^{\mathscr {K}}\big ({\varvec{\xi }}\big )\big )^2/M_{*},$ and then select the number of active functions that minimizes the MSE.

### Validation

We assessed and compared the PC expansions computed using either the OLS or OMP methods. Except for $\mathrm{pH}$, $\mathrm{pe}+\mathrm{pH},$ $\mathrm{S(VI)}$ and ${\mathrm{log}}_{10}\,{\mathrm{p}}_{{\mathrm{CO}}_2}$, a logarithmic transformation improved the surrogate approximations. Indeed, the log variables exhibited smoother dependences with respect to the uncertain input parameters than the original ones, and their use reduced the approximation errors of the original variables. In practice, the change of variable is trivial and consists of (i) building a PC expansion $v^{\mathscr {K}}({\varvec{\xi }})$ of $v({\varvec{\xi }}):=\log (u({\varvec{\xi }}))$ using the set $\mathscr {V}$ of logarithmically transformed outputs

14 $$\begin{aligned} \mathscr {V}:=\big \{v^{(m)}:=\log \big (u^{(m)}\big )\big \}, \end{aligned}$$

and (ii) applying the backward transformation to retrieve the original variables

15 $$\begin{aligned} u^{\mathscr {K}}({\varvec{\xi }}) :=\exp \left( v^{\mathscr {K}}({\varvec{\xi }})\right) . \end{aligned}$$

The PC expansions were built with a training set $\mathscr {X}$ of $M=10^{4}$ Monte-Carlo realizations and their errors were estimated using an independent validation set $\mathscr {X}_{*}$ of $M_{*}=10^4$ Monte-Carlo realizations. The accuracy of four PC expansions were compared with three obtained with the OLS method in which different maximal degrees were used $d^{\circ }=1, 2, 3$, and one obtained with the OMP using a dictionary of $N_{\mathrm{b}}(5)=42504$ functions. The number of PC basis functions for the OLS method is $N_{\mathrm{b}}(1)=21$, $N_{\mathrm{b}}(2)=210$, $N_{\mathrm{b}}(3)=1540$ while the number of active functions retained in the OMP method depends on the QoI and is reported in Table 3.

**Table 3:** Number of terms retained in the OMP method for each QoI.

QoI $\#$	1	2	3	4	5	6	7	8	9	10	11	12	13
Small range case	320	530	820	860	380	460	1130	420	1210	640	1170	960	190
Large range case	320	490	640	790	530	460	820	370	780	820	890	900	180

**Figure 1:**
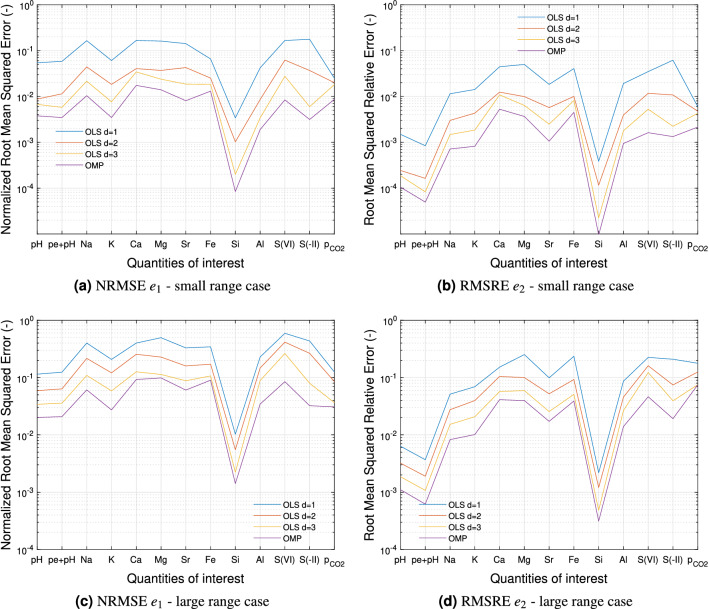
Validation errors for different PC expansions.

Two error metrics were used to estimate the accuracy of the approximations (Fig. 1): the root mean squared error normalized by the empirical variance $\widehat{\mathbb {V}}_{\mathscr {X}_{*}}(\cdot )$ of the QoI,

16 $$\begin{aligned} e_1 := \left[ \frac{1}{M_{*}}\sum _{{\varvec{\xi }}\in \mathscr {X}_{*}} \frac{\left( u\big ({\varvec{\xi }}\big )-u^{\mathscr {K}}\big ({\varvec{\xi }}\big )\right) ^2}{\widehat{\mathbb {V}}_{\mathscr {X}_{*}}(u)}\right] ^{1/2}, \end{aligned}$$

and the root mean squared relative error,

17 $$\begin{aligned} e_2 := \left[ \frac{1}{M_{*}} \sum _{{\varvec{\xi }}\in \mathscr {X}_{*}} \left( \frac{u\big ({\varvec{\xi }}\big )-u^{\mathscr {K}}\big ({\varvec{\xi }}\big )}{u\big ({\varvec{\xi }}\big )}\right) ^2\right] ^{1/2}. \end{aligned}$$

The global normalization of error $e_1$ allows to express the approximation error of the QoI in comparison with its uncertainty level whereas the local normalization of error $e_2$ is suitable when the approximation error and/or the QoI have different magnitudes across the parametric domain. The error levels obtained for the large range case were higher than for the small range case (roughly one order of magnitude) because large variations of input parameters induced more complex dependencies in geochemical reactions. As expected, the errors associated with the OLS method decreased when the maximal degree increased since the addition of higher order terms improved the approximations of the stochastic nonlinearities. A further increase of the maximal degree was not an option to reduce the error because the number of basis functions $N_{\mathrm{b}}(4)=8855$ was too close to the sample size $M=10^4$, thereby producing an ill-conditioned matrix $A^\top A$ in (11). On the contrary, the PC expansions obtained by the OMP method exhibited a higher accuracy and a lower number of terms (Table 3). Therefore, in subsequent analyses, we used the OMP surrogate models for which the error level was at most $1\%$ for $e_1$ and $0.5\%$ for $e_2$ in the small range case and $10\%$ for $e_1$ and $7\%$ for $e_2$ in the large range case. Lastly, we note that the input parameters distributions can be changed retroactively on a subset of the parametric domain provided that the surrogate model error is sufficiently low over this subset.

## Results and discussion

A direct exploitation of the PC coefficients was not feasible because of the logarithmic transformation. Statistical information were then derived promptly from extensive samplings of the surrogate models. We processed each QoI individually by studying their moments and marginal distributions. We then computed the correlations and plotted the joint distributions of the most correlated pairs of QoI. In closing, a global sensitivity analysis was carried out in order to rank the contribution of the uncertain input parameters onto the variance of each QoI.

### Moments

The empirical estimators of the mean $\mu$, the standard deviation $\sigma$ and the coefficient of variation $c_\mathrm{v}=\sigma /\mu$ of each QoI (Table 4) were obtained from a set $\mathscr {Y}$ of $N=10^6$ Monte-Carlo realizations of the surrogate models,

18 $$\begin{aligned} \widehat{\mu } = \widehat{\mathbb {E}}(u^{\mathscr {K}}) := \frac{1}{N} \sum _{{\varvec{\xi }}\in \mathscr {Y}} u^{\mathscr {K}}\left( {\varvec{\xi }}\right) \quad \text {and}\quad \widehat{\sigma }:= \left[ \frac{1}{N-1} \sum _{{\varvec{\xi }}\in \mathscr {Y}}\left( u^{\mathscr {K}}\left( {\varvec{\xi }}\right) - \widehat{\mu } \right) ^2\right] ^{1/2}. \end{aligned}$$

For most QoI, mean values and standard deviations had the same characteristics as the uncertain input parameters for the large and small range cases, i.e. the means were roughly identical while the standard deviations were multiplied by a factor 2. Iron and Aluminum concentrations were an exception because their mean were respectively 4 (Fe) and 1.7 (Al) times higher for the large range case than for the small range case. The ratio is 13 (Fe) and 3.5 (Al) for their standard deviation. Except for Al in the small range case, their standard deviations were larger than their mean values, pointing out a high dependence of $\mathrm{Al}$ and $\mathrm{Fe}$ concentrations to input model parameters variations. Low concentration of these two elements and a tight coupling of solubility controls exerted by two mineral phases, Ripidolite and Illite, for which the chosen uncertainty on solubility products were the highest, explained these findings (Table 2). On the contrary, pH values were remarkably stable despite the complex coupled control on this parameter exerted by many phases in the system^5^, thus showing a strong thermodynamic buffering of this parameter by the mineralogical assemblage.

**Table 4:** Empirical mean $\widehat{\mu }$, standard deviation $\widehat{\sigma }$, skewness $\widehat{s}$, kurtosis $\widehat{k}$ and coefficient of variation $\widehat{c_\mathrm{v}}$ estimated with $10^6$ realizations of the surrogate models.

QoI	Small range case					Large range case				
	$\widehat{\mu }$	$\widehat{\sigma }$	$\widehat{s}$	$\widehat{k}$	$\widehat{c_\mathrm{v}}\ [\%]$	$\widehat{\mu }$	$\widehat{\sigma }$	$\widehat{s}$	$\widehat{k}$	$\widehat{c_\mathrm{v}}\ [\%]$
$\mathrm{pH}$	7.17	0.20	$-0.004$	2.66	3	7.19	0.39	$-0.03$	2.68	5
$\mathrm{pe}+\mathrm{pH}$	4.20	$6.13\cdot 10^{-2}$	0.043	2.79	1.5	4.20	$1.26\cdot 10^{-1}$	0.17	2.97	3
${\mathrm{Na}}^{+}$	$4.29\cdot 10^{-2}$	$3.05\cdot 10^{-3}$	0.2	2.93	7	$4.43\cdot 10^{-2}$	$7.07\cdot 10^{-3}$	0.94	5.85	16
${\mathrm{K}}^{+}$	$1.04\cdot 10^{-3}$	$2.50\cdot 10^{-4}$	0.38	2.27	24	$1.17\cdot 10^{-3}$	$5.75\cdot 10^{-4}$	0.92	3.85	49
${\mathrm{Ca}}^{2+}$	$8.46\cdot 10^{-3}$	$2.21\cdot 10^{-3}$	0.52	3.16	26	$9.33\cdot 10^{-3}$	$4.80\cdot 10^{-3}$	1.48	7.89	51
${\mathrm{Mg}}^{2+}$	$5.91\cdot 10^{-3}$	$2.14\cdot 10^{-3}$	0.62	3.13	36	$6.68\cdot 10^{-3}$	$5.06\cdot 10^{-3}$	2.61	21.0	76
${\mathrm{Sr}}^{2+}$	$2.10\cdot 10^{-4}$	$2.87\cdot 10^{-5}$	0.45	3.08	14	$2.17\cdot 10^{-4}$	$6.42\cdot 10^{-5}$	1.05	4.95	30
$\mathrm{Fe}$	$4.59\cdot 10^{-5}$	$5.48\cdot 10^{-5}$	3.33	20.0	119	$1.85\cdot 10^{-4}$	$6.99\cdot 10^{-4}$	13.1	319.0	378
$\mathrm{Si}$	$1.83\cdot 10^{-4}$	$2.11\cdot 10^{-5}$	0.14	1.83	12	$1.87\cdot 10^{-4}$	$4.29\cdot 10^{-5}$	0.28	1.89	23
$\mathrm{Al}$	$9.34\cdot 10^{-8}$	$5.25\cdot 10^{-8}$	1.05	3.83	56	$1.55\cdot 10^{-7}$	$1.83\cdot 10^{-7}$	2.38	10.9	117
$\mathrm{S(VI)}$	$1.46\cdot 10^{-2}$	$3.00\cdot 10^{-3}$	0.32	2.84	21	$1.66\cdot 10^{-2}$	$8.63\cdot 10^{-3}$	2.43	18.3	52
$\mathrm{S(-II)}$	$7.50\cdot 10^{-10}$	$3.93\cdot 10^{-10}$	1.80	8.15	52	$1.31\cdot 10^{-9}$	$1.84\cdot 10^{-9}$	5.65	62.2	141
${\mathrm{log}}_{10}\,{\mathrm{p}}_{{\mathrm{CO}}_2}$	$-2.07$	0.41	$-0.014$	2.66	$-20$	$-2.11$	0.79	$-0.058$	2.62	$-38$

### Marginal distributions

**Figure 2:**
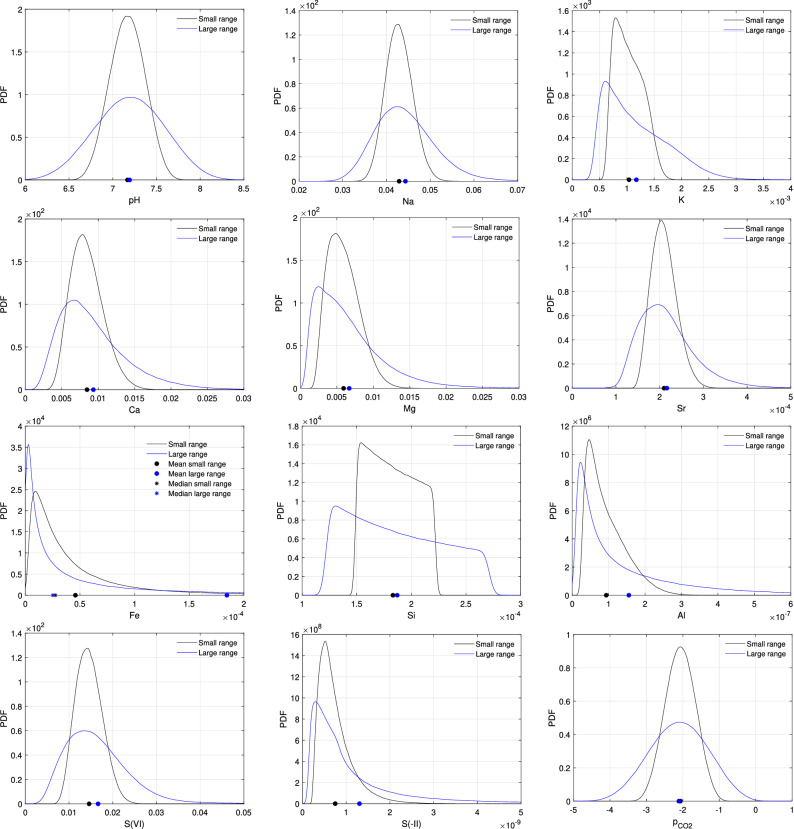
Marginal distributions and means of the QoI for the small range case (black curves) and the large range case (blue curves) estimated with $10^6$ realizations of the surrogate models and the kernel density estimation method^32^.

Small range case results exhibited three types of empirical marginal distribution profiles (Fig. 2): bell-shaped distributions for $\mathrm{pH}$, $\mathrm{pe}+\mathrm{pH}$ (not shown), ${\mathrm{Na}}^{+}$, ${\mathrm{K}}^{+}$, ${\mathrm{Ca}}^{2+}$, ${\mathrm{Mg}}^{2+}$, ${\mathrm{Sr}}^{2+}$, $\mathrm{S(VI)}$, and ${\mathrm{log}}_{10}\,{\mathrm{p}}_{{\mathrm{CO}}_2}$; right-skewed distributions for $\mathrm{Al}$, $\mathrm{S(-II)}$, and $\mathrm{Fe}$; and a piecewise linear distribution for $\mathrm{Si}$. Large range case results led to a flattening of the distributions (except for $\mathrm{Fe}$), which was coherent with variances increase. The shape of a distribution can be described by skewness *s* and kurtosis *k* that are defined as the third and fourth standardized moments, respectively. The empirical estimators of *s* and *k*, indicated in Table 4, are

19 $$\begin{aligned} \widehat{s} := \frac{\widehat{\mathbb {E}}\left[ \left( u^{\mathscr {K}}\left( {\varvec{\xi }}\right) - \widehat{\mu } \right) ^3\right] }{\widehat{\sigma }^3} \quad \text {and}\quad \widehat{k} := \frac{\widehat{\mathbb {E}}\left[ \left( u^{\mathscr {K}}\left( {\varvec{\xi }}\right) - \widehat{\mu } \right) ^4\right] }{\widehat{\sigma }^4}. \end{aligned}$$

The skewness of a distribution measures its asymmetry and a distribution is commonly said to be fairly symmetrical if $|s|\le 1/2$, moderately skewed if $1/2\le |s|\le 1$ and highly skewed if $|s|\ge 1$. In the small range case, we observed a slight asymmetry for ${\mathrm{Ca}}^{2+}$ and ${\mathrm{Mg}}^{2+}$ and a high asymmetry for $\mathrm{Al}$, $\mathrm{S(-II)}$, and $\mathrm{Fe}$. In the large range case, the asymmetry became important for all the QoIs except for $\mathrm{pH}$, $\mathrm{pH}+\mathrm{pe}$, $\mathrm{Si}$ and ${\mathrm{log}}_{10}\,{\mathrm{p}}_{{\mathrm{CO}}_2}$. The kurtosis of a distribution measures the combined weight of the tails relative to the rest of the distribution. It is common to compare the kurtosis to 3 which is the kurtosis of a normal distribution; a high kurtosis ($k>3$) indicates heavy tails while low kurtosis ($k<3$) denotes light tails. In the small range case, the kurtosis is between 2 and 4 except for Fe and $\mathrm{S(-II)}$ which have strong heavy-tailed distributions and $\mathrm{Si}$ due to its piecewise linear distribution. In the large range case, we observed that the kurtosis of each distribution increases substantially (except for $\mathrm{pH}$, $\mathrm{pH}+\mathrm{pe}$, $\mathrm{Si}$ and ${\mathrm{log}}_{10}\,{\mathrm{p}}_{{\mathrm{CO}}_2}$), meaning that the heaviness of the tails grows in importance. We noted that $\mathrm{Fe}$ was the only quantity of which the distribution was more peaked for the larger parametric domain; the mean values obtained with each of these cases were significantly different but the medians were very close (Fig. 2).

### Linear correlations

Linear correlation between two random variables *u* and *v* were measured with the Pearson’s correlation coefficient $r(u,v)\in [-1,1]$ defined as follows

20 $$\begin{aligned} r(u,v):=\frac{{\mathbb C}\mathrm{ov}(u,v)}{\sigma (u)\sigma (v)}, \end{aligned}$$

where ${\mathbb C}\mathrm{ov}(u,v):=\mathbb {E}\left[ (u-\mathbb {E}(u))(v-\mathbb {E}(v))\right]$ is the covariance between *u* and *v*. The square of Pearson’s coefficient is the coefficient of determination $R^2(u,v):=r(u,v)^2\in [0,1]$, which represents the percentage of variation of *u* due to a linear variation of *v*.

**Figure 3:**
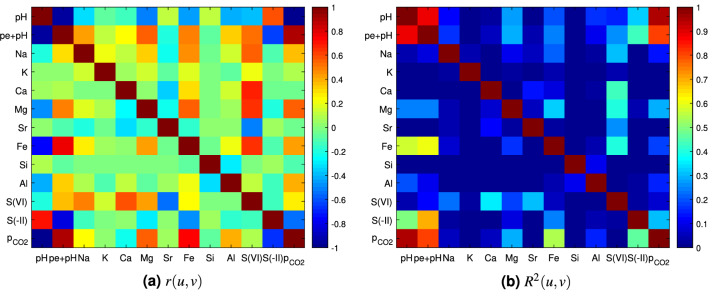
Matrices of correlation coefficient *r*(*u*, *v*) and coefficient of determination $R^2(u,v)$ for the small range case (lower part of matrices) and the large range case (upper part of matrices) estimated with $10^6$ realizations of the surrogate models.

Empirical estimates of *r*(*u*, *v*) and $R^2(u,v)$ are plotted on Fig. 3 in which the lower (resp. upper) parts of the matrices correspond to the small (resp. large) range case. Three pairs presented a particularly strong correlation regardless of the parametric domain size: the pairs $(\mathrm{pH},\mathrm{pe}+\mathrm{pH})$ and $(\mathrm{pH},{\mathrm{log}}_{10}\,{\mathrm{p}}_{{\mathrm{CO}}_2})$ were negatively correlated with $R^2=88\%$ and $R^2=97\%$ respectively, whereas the pair $(\mathrm{pe}+\mathrm{pH},{\mathrm{log}}_{10}\,{\mathrm{p}}_{{\mathrm{CO}}_2})$ was positively correlated with $R^2=80\%$. The $(\mathrm{pH},{\mathrm{log}}_{10}\,{\mathrm{p}}_{{\mathrm{CO}}_2})$ pair correlation can be understood by noting that the standard deviation of ${\mathrm{Ca}}^{2+}$ concentration (Table 2) was small compared to its mean value (Table 1) and that the ${\mathrm{log}}_{10}\,{\mathrm{p}}_{{\mathrm{CO}}_2}$ value is directly related to $\mathrm{pH}$ by the Calcite equilibrium reaction. The correlation in the pair $(\mathrm{pH},\mathrm{pe}+\mathrm{pH})$ cannot be explained by the known negative correlation of the pair $(\mathrm{pe},\mathrm{pH})$ at constant dioxygen or dihydrogen fugacity through corresponding Nernst’s equation, which results in a $-1$ slope in the $\mathrm{pe}-\mathrm{pH}$ diagram representation: the QoI transformation from $\mathrm{pe}$ to $\mathrm{pe}+\mathrm{pH}$ was indeed meant to suppress this correlation. Consequently, the observed negative correlation must be attributed to particular equilibrium reactions. Goethite equilibrium, the reaction of which results in a $-3$ slope in a $\mathrm{pe}-\mathrm{pH}$ diagram, may explain the observed correlation.

Four pairs had a moderate correlation for the small range case that significantly decreased for the large range case: the pairs $(\mathrm{pH},\mathrm{Fe})$ and $(\mathrm{pe}+\mathrm{pH},\mathrm{S(-II)})$ were negatively correlated with $R^2=57\%$ and $R^2=70\%$. The correlation is well explained by the sensitivity of $\mathrm{S(-II)}$ and $\mathrm{Fe}$ concentration to redox conditions. The pairs $(\mathrm{Fe},{\mathrm{log}}_{10}\,{\mathrm{p}}_{{\mathrm{CO}}_2})$ and $(\mathrm{pe}+\mathrm{pH},\mathrm{Fe})$ were positively correlated with $R^2=53\%$ and $R^2=60\%$. Inversely, the correlation of some pairs involving $\mathrm{S(VI)}$ was higher for the large range case: $(\mathrm{Fe},\mathrm{S(VI)})$, $({\mathrm{Ca}}^{2+},\mathrm{S(VI)})$, and $({\mathrm{Mg}}^{2+},\mathrm{S(VI)})$ with $R^2=46\%$, $R^2=42\%$, and $R^2=40\%$, respectively (instead of $0.5\%$, $35\%$, and $19\%$ for the small range case). These observations can be related to the charge balance requirement in aqueous solution during the calculation. In the model, ${\mathrm{Na}}^{+}$ and ${\mathrm{Cl}}^{-}$ total concentrations (aqueous + exchange) are stabilized at their final values before the reaction step with minerals. Mineral phases exert no further control on their concentrations. Hence, a variation of $\mathrm{S(VI)}$ concentration, which is the second major anion in solution, must be compensated by an equivalent variation of cations concentrations to fulfill solution electroneutrality. This compensation is mostly achieved by ${\mathrm{Ca}}^{2+}$, ${\mathrm{Mg}}^{2+}$, and $\mathrm{Fe}$ because ${\mathrm{Na}}^{+}$ total concentration is fixed by the amount available on the cation exchanger, and because ${\mathrm{Sr}}^{2+}$ concentration is controlled by Celestite solubility, which is itself linked to $\mathrm{S(VI)}$ concentration.

### Bivariate distributions

**Figure 4:**
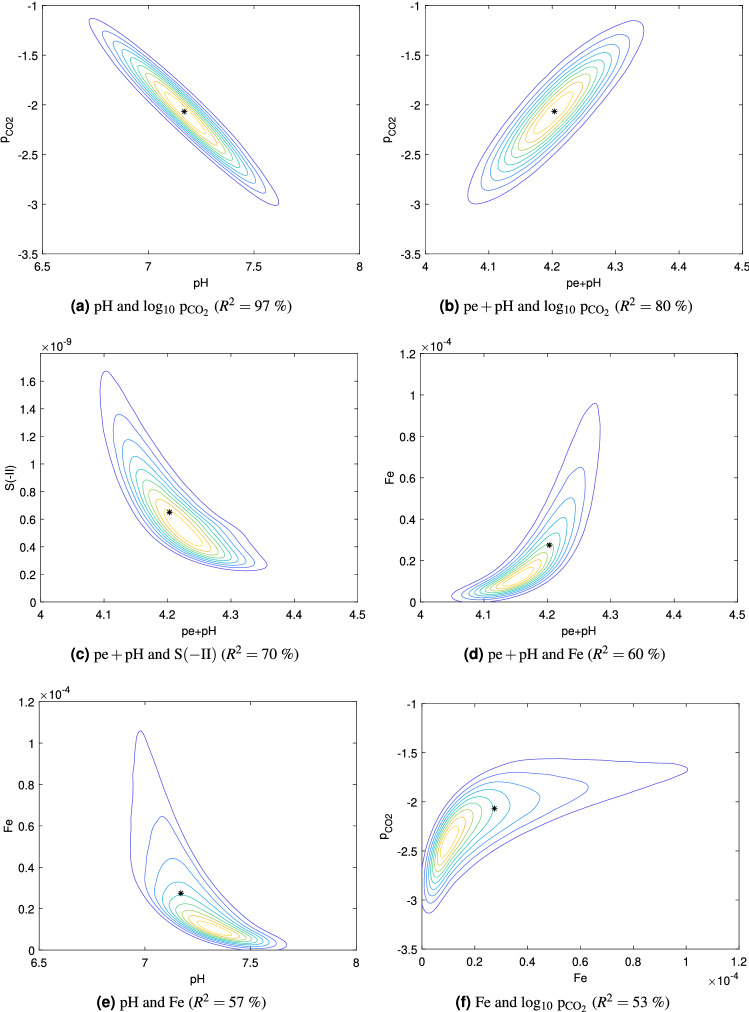
Isolines of bivariate distributions and medians (asterisks) estimated with $10^6$ realizations of the surrogate models and the kernel density estimation method for the most correlated pairs of QoI (small range case). The plots are ordered according to the value of $R^2$.

The shapes of the isolines contours of the most correlated pairs of QoI ($R^2>50\%$) were clearly consistent with the sign of the correlation coefficient (Fig. 4), namely negative for the pairs $(\mathrm{pH},{\mathrm{log}}_{10}\,{\mathrm{p}}_{{\mathrm{CO}}_2})$, $(\mathrm{pH},\mathrm{Fe})$, $(\mathrm{pe}+\mathrm{pH},\mathrm{S(-II)})$ and positive for the pairs $(\mathrm{pe}+\mathrm{pH},{\mathrm{log}}_{10}\,{\mathrm{p}}_{{\mathrm{CO}}_2})$, $(\mathrm{Fe},{\mathrm{log}}_{10}\,{\mathrm{p}}_{{\mathrm{CO}}_2})$, $(\mathrm{pe}+\mathrm{pH},\mathrm{Fe})$. Also, the pairs $(\mathrm{pH},{\mathrm{log}}_{10}\,{\mathrm{p}}_{{\mathrm{CO}}_2})$ and $(\mathrm{pe}+\mathrm{pH},{\mathrm{log}}_{10}\,{\mathrm{p}}_{{\mathrm{CO}}_2})$ followed a bivariate normal distribution whereas the other pairs exhibited more complex asymmetrical distributions. Isolines of the pair $(\mathrm{pH},\mathrm{pe}+\mathrm{pH})$ had the same pattern as those of the pair $(\mathrm{pH},{\mathrm{log}}_{10}\,{\mathrm{p}}_{{\mathrm{CO}}_2})$ (not shown).

### Global sensitivity analysis

An essential aspect of uncertainty propagation is the global sensitivity analysis^33,34^, which quantifies the relative contribution of each uncertain input parameter (or group of input parameters) to the variance of the QoI. This analysis across the whole parametric domain should not be confused with local sensitivity analysis^35^, which estimates the effect of small perturbations around specific input values by means of the partial derivatives of the model. The global sensitivity analysis was based on the decomposition of the total variance^36^ into $2^{N}-1$ terms ($N=19$ in this study), as follows

21 $$\begin{aligned} \mathbb {V}(u)= \sum _{i=1}^N \mathbb {V}_i + \sum _{i<j}\mathbb {V}_{ij}+\cdots +\mathbb {V}_{1\ldots N}, \end{aligned}$$

where $\{\mathbb {V}_i\}$ are the first-order interaction terms, $\{\mathbb {V}_{ij}\}$ the second order terms, and so on. Of particular interest are the $\mathbb {V}_i$ which measure the own effects of the input parameter $\xi _i$ on the output variance. Typically, these effects are normalized by the total variance defining the first-order sensitivity indices $S_i$ by

22 $$\begin{aligned} S_i:=\frac{\mathbb {V}_i}{\mathbb {V}}. \end{aligned}$$

**Figure 5:**
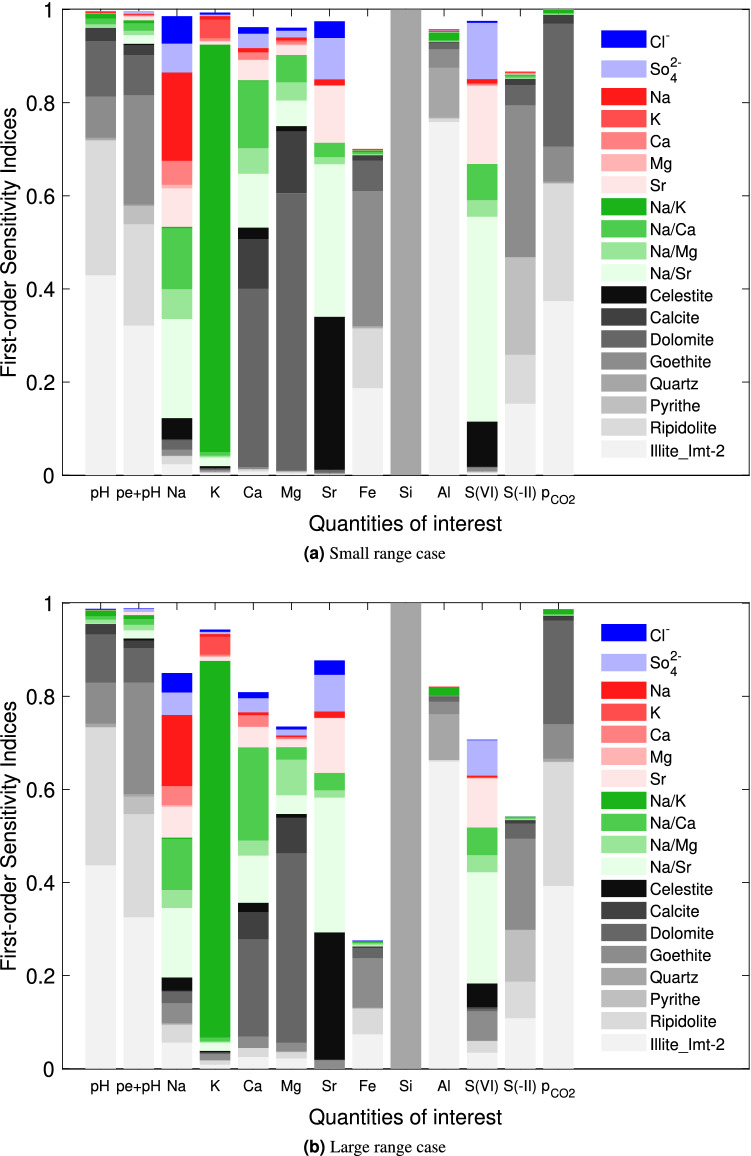
First-order sensitivity indices $S_i$ (own effects). The color depends on the input parameter category: blue for the leached parameters, red for the exchanged cations, green for the selectivity coefficients, and gray for the solubilities. The color shade varies within each category.

The first-order sensitivity indices were estimated from the Monte-Carlo pick-freeze algorithm^33,37^, which requires a sample of size *M* of the input variables (Fig. 5). For a given case, the number of surrogate model evaluations was $M(N+1)=2\cdot 10^7$ for each QoI. A sum of the first-order indices close to one is representative of low interactions between parameters and of an essentially additive model. Interaction effects were minor for the small range case (except for $\mathrm{Fe}$ and $\mathrm{S(-II)}$), but increased significantly for the large range case. The first-order sensitivity indices of eight quantities, $\mathrm{pH}$, $\mathrm{pe}+\mathrm{pH}$, ${\mathrm{Mg}}^{2+}$, $\mathrm{Fe}$, $\mathrm{Si}$, $\mathrm{Al}$, $\mathrm{S(-II)}$, ${\mathrm{log}}_{10}\,{\mathrm{p}}_{{\mathrm{CO}}_2}$ were mainly governed by solubilities, while four other quantities, ${\mathrm{Na}}^{+}$, ${\mathrm{Ca}}^{2+}$, ${\mathrm{Sr}}^{2+}$, $\mathrm{S(VI)}$, depended on the four input categories. In addition, three QoIs were strongly dependent on a single parameter: $\log K_{\mathrm{ex}}^{{\mathrm{Na}}^{+}/{\mathrm{K}}^{+}}$ for ${\mathrm{K}}^{+}$ consistently with the known control of ${\mathrm{K}}^{+}$ concentration by cation exchange reactions in clay minerals rich systems^38^; quartz for $\mathrm{Si}$ consistently with the negligible variation of quartz solubility product and with $\mathrm{Si}$ aqueous speciation in the explored range of pH variations; and Illite for $\mathrm{Al}$ consistently with the fact that only Illite and Ripidolite react with $\mathrm{Al}$.

## Conclusion

Our uncertainty propagation study using surrogate models proved to be successful in analyzing the sensitivity of a reference pore water geochemical model to its various input parameters. The results, and validation with direct Monte-Carlo simulations, show that sparse polynomial chaos are well-adapted to approximate the quantities of interest. Most significant correlations and anti-correlations were tractable from geochemical constraints, giving confidence in the overall analysis. The method makes it possible not only to quantify the uncertainties of the quantities of interest for future performance evaluation calculations, but also to identify the main influential input parameters. This latter information is particularly valuable to guide further research efforts in view of reducing uncertainties on specific aspects of performance assessment analyses. Because pore water chemistry influences many important parameters such as radionuclides transport and retardation by adsorption and precipitation, uncertainty analyses of reactive transport modeling outcomes would certainly benefit from a coupling with our surrogates models to decipher uncertainties in adsorption models predictions, and to speed up calculations in fully coupled approaches.

## Supplementary Information

Supplementary Information 1.

Supplementary Information 2.

## Data Availability

All data generated or analysed during this study are included in this published article and its supplementary information files.
