# Supplementary material for: Uncertainty propagation in pore water chemical composition calculation using surrogate models
Source: Sci Rep. 2022 Sep 5;12:15077. doi: 10.1038/s41598-022-18411-5 (PMC9445106; doi:10.1038/s41598-022-18411-5)
Supplement: Supplementary file 1 — Supplementary Information 1. [file 41598_2022_18411_MOESM1_ESM.pdf]

Supplementary Information

Pore water composition model description

Our geochemical model (Fig. 1) is built on Gibbs' phase rule and assumes a local thermodynamic equilibrium of water composition with a set of minerals present in the claystone and showing no evidence of alteration[1]. Gibbs' phase rules states that the number of degrees of freedom in a system is equal to the number of independent components, minus the number of phases in equilibrium with each other, plus two because of related pressure and temperature parameters. Consequently, our modeling approach consists in finding a number of mineral constraints equal to the number of independent chemical components, while pressure and temperature are set at the measured *in situ* values. Concentrations of conservative ions such as chloride (Cl^-) or sodium (Na^+) are not controlled by equilibrium with mineral phases, but are obtained from leached anions (for Cl^-) and exchanged cations (for Na^+) measurements on core sample. The last constraint is the presence of water as the solvent phase, which corresponds to an electroneutrality condition in the model.

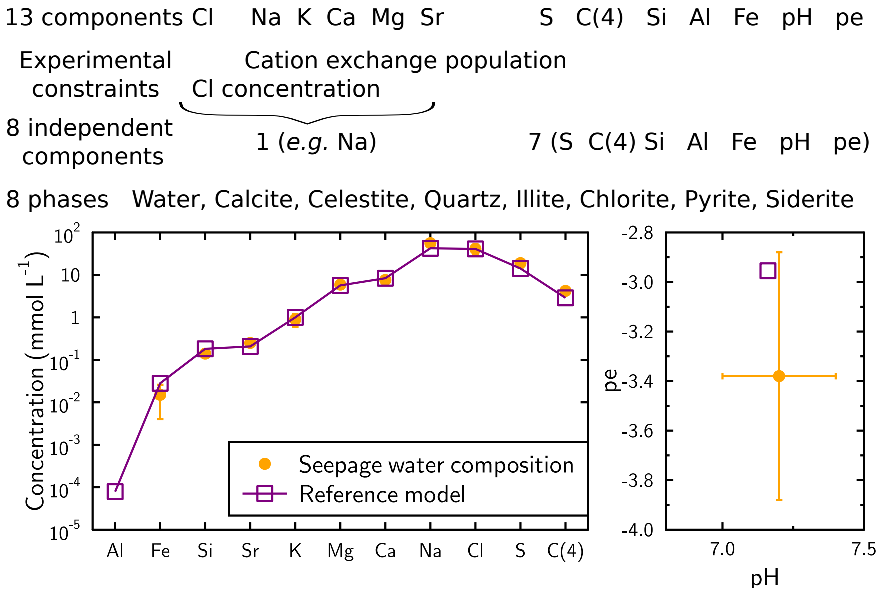

Figure 1. Schematic explanation of the thermodynamic model used to calculate the pore water chemical composition in COx claystone. Simulated values correspond to the result of the PHREEQC model file provided in the supplementary data file. Seepage water composition was taken from[2].

Orthogonal matching pursuit procedure

Sparse approximation theory consists in finding parsimonious solutions of underdetermined linear systems. The minimization problem is formally written as

$$\min_{\boldsymbol{u}} \|\boldsymbol{u}\|_0 \quad \textit{subject to} \quad \mathbb{y} = D\boldsymbol{u}, \tag{1}$$

where $\mathbb{y} \in \mathbb{R}^M$ is the vector of observations (or simulations), $D \in \mathbb{R}^{M,N}$ is a dictionary of N functions (also called atoms), and $\boldsymbol{u} \in \mathbb{R}^N$ is a sparse vector with s nonzero coefficients, with $s < M \ll N$. The ℓ_0 pseudo-norm $\|\boldsymbol{u}\|_0$ is defined as $\#\{i, u_i \neq 0, 1 \leq i \leq N\}$ and counts the nonzero coefficients of $\boldsymbol{u} = [u_i]$. Although Problem (1) is known to be NP-hard, its solution can be estimated using sparse recovery algorithms and we refer to[3] for a review of these algorithms. In particular, the matching pursuit[4] and its variants form a set of greedy algorithms, making a local optimal selection at each iteration in hopes of finally converging to the global optimum solution. Specifically, the matching pursuit is a forward stepwise regression approach: the approximation starts from zero and is enriched at each iteration with the function of D the most correlated with the current residual. A popular extension of matching pursuit is its orthogonal version in which the residual is orthogonal to the span of all the functions already selected.

The successive steps of the orthogonal matching pursuit procedure are outlined in Algorithm 1. The input data are the vector of observations $\mathbb{y}$ as well as the dictionary of functions D whose every column $\mathbb{d}_i$ must have a unit ℓ_2-norm. In our application, the dictionary corresponds to the matrix of PC basis functions evaluated at the training sample points and normalized accordingly. In the initialization step, the residual $\mathbb{r}^0$ is set to the vector of observations and the set of active function

Γ^0 is empty. During each iteration, the index γ^k of the function the most correlated with the residual is selected (line 3) and added to the set Γ^k of active basis functions (line 4). An ordinary least squares method is then applied to estimate the spectral coefficients $\boldsymbol{u}^k$ associated to the active set (line 5). To mitigate the computational complexity of this step, the least-squares solution is computed by using the Schur complement to update the information matrix inverse whenever a new regressor is added. The next steps of the algorithm are the computation of the vector approximation $\mathbb{u}^k$ (line 6) and the residual $\mathbb{r}^k$ (line 7). The iterations are continued until a stopping criteria, concerning either a given number of active basis functions or an error threshold, is achieved. The output data are the set of active basis functions and the spectral coefficients.

Algorithm 1 Orthogonal matching pursuit algorithm

Input: $\mathbb{u}$ and $D = [\mathbb{d}_1, \cdots, \mathbb{d}_N]$

1: $\mathbb{r}^0 = \mathbb{u}$, $\Gamma^0 = \emptyset$, $k = 1$ ▷ Initialization

2: **repeat**

3: $\gamma^k = \arg\max_{j \notin \Gamma^{k-1}} \left(\left| \mathbb{d}_j^\top \mathbb{r}^{k-1} \right| \right)$, ▷ Select new index

4: $\Gamma^k = \Gamma^{k-1} \cup \{\gamma^k\}$, ▷ Update index set

5: $\left(A^k\right)^\top A^k \boldsymbol{u}^k = \left(A^k\right)^\top \mathbb{u}$, ▷ Parameter vector estimation

6: $\mathbb{u}^k = A^k \boldsymbol{u}^k$, ▷ Approximation computation

7: $\mathbb{r}^k = \mathbb{u} - \mathbb{u}^k$, ▷ Residual computation

8: $k \leftarrow k + 1$

9: **until** Stopping criteria is satisfied

Output: Γ^k and $\boldsymbol{u}^k$

References

1. Lerouge, C. *et al.* Mineralogical and isotopic record of biotic and abiotic diagenesis of the Callovian-Oxfordian clayey formation of Bure (France). *Geochim. Cosmochim. Acta.* **75**, 2633–2663 (2011).

2. Vinsot, A., Mettler, S. & Wechner, S. In situ characterization of the Callovo-Oxfordian pore water composition. *Phys. Chem. The Earth* **33**, S75–S86 (2008).

3. Crespo Marques, E., Maciel, N., Naviner, L., Cai, H. & Yang, J. A review of sparse recovery algorithms. *IEEE Access* **7**, 1300–1322, DOI: 10.1109/ACCESS.2018.2886471 (2019).

4. Mallat, S. & Zhang, Z. Matching pursuits with time-frequency dictionaries. *IEEE Trans. Signal Process.* **41**, 3397–3415 (1993).
